# Sweet 452 km – a report on the first type 1 diabetes patient to finish Double Ironman, a 30-hour endurance triathlon race

**DOI:** 10.3325/cmj.2013.54.306

**Published:** 2013-06

**Authors:** Pavao Vlahek, Siniša Car, Ivanka Ostroški

**Affiliations:** 1 Special Rehabilitation Hospital Varaždinske Toplice, Varaždinske Toplice, Croatia; 2 Varaždin General Hospital, Varaždin, Croatia

Scientific research of type 1 diabetes patients is often limited by ethical or technical reasons. Therefore, when people with diabetes decide to push their own limits and stretch the limits of our knowledge on their own, we can just observe and try to alleviate possible dangers. Here we would like to present a case of the first type 1 diabetes patient to safely complete the Double Ironman triathlon race. The race consisted of consecutive 7.6 km of swimming, 360 km of cycling, and 84.4 km of running.

## History and examination

The 27-year old patient has suffered from type 1 diabetes from his 6th year and used various insulin forms: Homologue+Homorap combination from age 6 to 11 years, Actrapid+Insulatard from age 9 to 21, and Novorapid+Lantus from the age of 22 till present. Before he started to compete in triathlons 3 years ago, the patient had had a history of obesity and badly regulated blood glucose level and calorie intake with frequent episodes of hypoglycemia.

After having entered a structured swimming, cycling, and running program he finished shorter triathlon distances and after 2 years in training completed the Ironman triathlon (3.8 km swimming, 180 km cycling, and 42.2 km running). This resulted not only in athletic achievement but also helped him to maintain normal body mass index, regulate blood glucose, and terminate hypoglycemic episodes. The usual insulin application was 25-30 IU/d administered by an insulin pen depending on calorie intake and training. The patient trained 2 hours on weekdays and up to 10 hours on weekend. The next goal was Double Ironman.

## The race report

**Figure 1:**
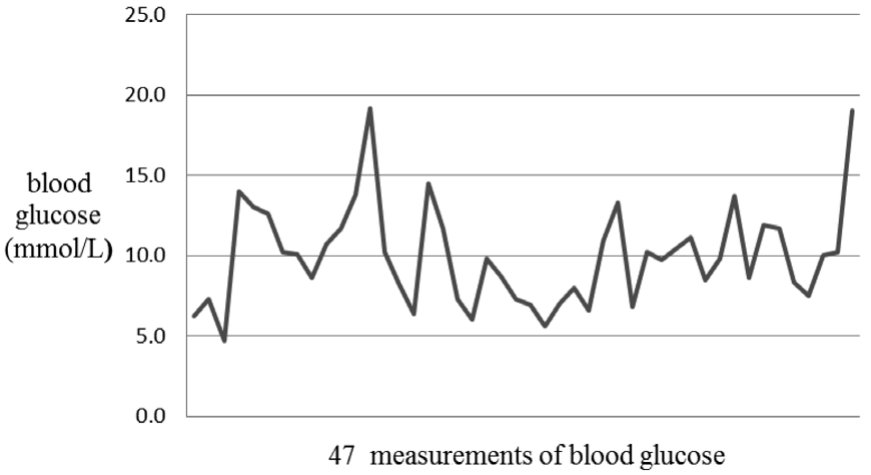
Blood glucose during the race.

During the 30 hours of race, blood glucose, calorie, and fluid intake were measured and insulin was applied. Complete blood count, and metabolic and biochemical parameters were measured 1 hour before the race, as well as 1 hour, 24 hours, and 7 days after the race. The race started at 16:00 with swimming. The air temperature was 29°C and the water temperature was 24°C. The cycling portion started at 19:27 and lasted until 10:15 next day, the air temperature staying around 20°C. The running portion started at 10:15 and finished at 21:10. During the day, the air temperature rose up to 35°C. Since scientific investigation had only secondary importance, measurements of blood glucose were taken at athlete’s will, 46 times. Blood glucose was measured approximately every 30 minutes during the swimming segment and every 45 to 90 minutes during cycling and running. During swimming, higher levels of blood glucose were maintained to avoid hypoglycemia (Figure 1). Calorie intake during the entire 29 hours and 15 minutes of racing was approximately 16 000 kcal in various foods and fluids, while the fluid intake was 23 L in the form of isotonic drinks, water, and sweetened beverages. During the race, only 18 IU of Novorapid was applied, primarily after hyperglycemic episodes during transition stops between swimming-cycling and cycling-running portions.

All the blood parameters 1 hour before the race were within the reference range. HbA1C measurement 2 days before the race was 5.5. One hour after the finish several parameters were elevated: leukocytes were 14.5 × 10^9^, bilirubin 23.3 µmol/L, urea 8.9 mmol/L, creatinine 110 µmol/L, CRP 18.1 mg/L, AST 146 IU/L, and CK 3234 IU/L. After 24 hours, only some parameters remained elevated: CRP was 14.5 mg/L, AST 197 IU/L, and CK 2479 IU/L. After 7 days, all the parameters were again within the reference range. The parameters were similar to those in a healthy person enduring a similar race.

## Conclusions

Although our patient showed that it was possible for a person with type 1 diabetes to participate in such a strenuous and long lasting event, we would not go so far as to conclude that ultra-endurance events and extreme physiological conditions are generally safe for people suffering from diabetes. Also, there is a lack of data about long-term consequences of such participation. There are only few reports on diabetes type 1 patients participating in endurance events. Most report participation in shorter races such as marathons (1-3) and emphasize dangers of hypoglycemia (4-7).

Despite dangers and obstacles, the potential benefit for a person with type 1 diabetes involved in endurance sports could be considerable. Athletes maintain a healthy lifestyle, closely monitor their blood glucose status, and serve as motivation for other people living with diabetes to involve in regular moderate exercise.
